# Central Integration of Canal and Otolith Signals is Abnormal in Vestibular Migraine: A Commentary

**DOI:** 10.3389/fneur.2014.00263

**Published:** 2015-01-06

**Authors:** Marcelo M. Valença

**Affiliations:** ^1^Neurology and Neurosurgery Unit, Federal University of Pernambuco and Hospital Esperança, Recife, Brazil

**Keywords:** vestibular migraine, headache, cerebellum, brainstem, pathophysiology

This is a very interesting and well-written article describing possible mechanisms that may contribute to the vestibular symptoms and motion intolerance that are most commonly seen in vestibular migraine. (1) The discussion in the article is cogent, based on the experimental results presented, exemplifying the idea of multiple potential interactions between migraine and the vestibular system, thus suggesting that the vertigo in vestibular migraine is multifactorial and may include peripheral (e.g., labyrinthine), as well as brain components (vestibular nuclei and *locus coeruleus*).

Vestibular migraine is said to be the second most frequent cause of vertigo and, in fact, appears to be the commonest cause of spontaneous episodic vertigo (2). In a study by Neuhauser and colleagues (3, 4), evaluating the presence of both lifetime migraine and vertigo, the authors found that a significant percentage of the migraineurs complained of vertigo. In addition, in most vestibular migraine sufferers, the symptomatology apparently occurs without the headache itself. Migraine headache attacks may also begin earlier in life than vestibular migraine symptoms appearance or, on the contrary, patients may report attacks of vestibular migraine years before the regular occurrence of migraine headaches (5). It is noteworthy that specific antimigraine drugs (triptans) when used to relieve the vestibular migraine symptoms may trigger headache (6). Taken together, these considerations lead us to presume that distinct mechanisms are at work in both vestibular and “typical” forms of migraine.

Even today, there is still much debate on the pathophysiology of migraine, particularly concerning the initial anatomical location in the brain, where a migraine attack is triggered – brain cortex vs. brainstem. The idea of also considering the cerebellum as another possible location of the initiation of a migraine crisis is intriguing. If this is indeed the case, the more caudal part of the cerebellar vermis, i.e., *nodulus* and uvula, would seem to be particularly involved in the mechanism linked to vestibular migraine. The presentation of vestibular symptoms without headache in migraineurs is a plausible example that strongly suggests the hypothesis of a cerebellar (or brainstem) origin of a vestibular migraine attack. On the other hand, cerebellar activation may occur later on, during the course of such an attack.

Neuroimaging studies have demonstrated that specific cerebellar lesions can be found in migraine patients (7, 8), in addition to periventricular white matter abnormalities (9). Brainstem lesions may also be detected in such patients. This indicates that migraine is not such a benign disease as previously believed (Figure 1) (10). Subclinical cerebellar dysfunction was reported in migraineurs (11). Does the accumulation of brain lesions throughout life alter the severity of the migraine disorder? This may be an explanation for the appearance of new symptoms in the same individual or could also be one of the causes of the transformation of an episodic migraine into a chronic one. The abovementioned considerations suggest that migraine disorders are linked to specific anatomical changes (12), which may be initially biochemical, and thus without neuroimaging findings, but in some migraineurs with a more severe form of the disease, or when some comorbid conditions are also present (e.g., dyslipidemia), “specific,” and significant abnormalities are occasionally encountered during a MRI study (7–10). This said, the concept that the migraine disorder harbored by a particular individual progresses throughout life in different forms of presentation, particularly in females, owing to hormonal fluctuations, namely in childhood with its migraine equivalents, becoming more apparent in the menarche period, attenuating during pregnancy or lactation, and becoming oligosymptomatic or absent in the post-menopausal period.

**Figure 1:**
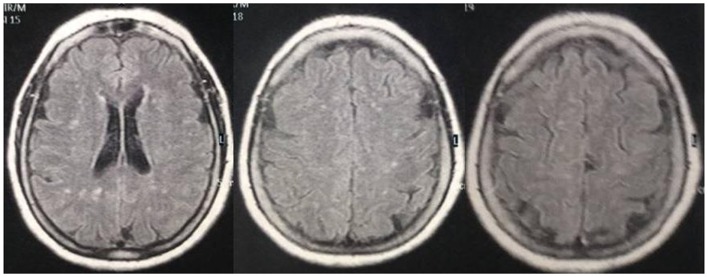
**MRI of a 69-year-old woman with a long history of migraine attacks**. Note the white matter abnormalities, frequently encountered in migraineurs.

It is common knowledge that the clinical phenomenology of migraine disorders covers a spectrum ranging from a mild to an extremely severe and incapacitating form, both between attacks in the same individual and among different subjects. The migraine syndrome consists of a variety of symptoms, such as auras, autonomic manifestations, pain, auditory and vestibular complaints, etc., and each of these may involve specific activation of distinct neuronal networks in the brain or peripheral nervous system and its innervated tissues, such as those of the vascular system (13). We are still largely ignorant of exactly how and why this occurs.

Another point is that there may be different forms of migraine, genetically distinct but with a similar clinical presentation, which we, as headache specialists, are pooling as a single disease entity. Owing to this multiplicity of manifestations or possible grouping together of several distinct “migraine disorders,” the precise physiopathogenesis has still to be clarified. The study being published by King and coworkers selected a highly specific population to evaluate the physiopathology of vestibular migraine, focusing on one particular subset of a migraine population.

In conclusion, vestibular migraine is a relatively common clinical condition not fully understood that has still to be recognized as such by the medical community in general.

## Conflict of Interest Statement

The author declares that the research was conducted in the absence of any commercial or financial relationships that could be construed as a potential conflict of interest.
